# Supplementary material for: Human Genome Polymorphisms and Computational Intelligence Approach Revealed a Complex Genomic Signature for COVID-19 Severity in Brazilian Patients
Source: Viruses. 2023 Feb 28;15(3):645. doi: 10.3390/v15030645 (PMC10059592; doi:10.3390/v15030645)
Supplement: Supplementary file 1 [file viruses-15-00645-s001.zip › S2. Metrics for all evaluated classifiers.pdf]

Supplementary Material – Results for all evaluated classifiers

This table shows the metrics for all evaluated classifiers, over test set data in the Patient Prognosis phase (item 2.5 of manuscript) after feature selection phase. Inputs to each model are: rs1990760 (*IFIH1*), rs2161525 (*DC-SIGNR*), rs4251513 (*IRAK4*), rs3024498 (*IL10*), rs2508450 (*IL10RA*), rs16923189 (*PD-L2*), rs17804441 (*PD-L1*), rs1051922 (*IFNB1*), rs12340866 (*JAK2*), rs3771300 (*STAT1*), rs303215 (*IFIT1*), and rs17622656 (*IRF1*).

**Metrics**	**Logistic Regression**	**KNN**	**DT**	**SVM – RBF Kernel**	**SVM – Linear Kernel**
Test Accuracy	0.8500	0.5500	0.7000	0.7500	0.8500
Specificity	0.8000	0.5000	0.5000	0.7000	0.9000
Sensitivity	0.9000	0.6000	0.9000	0.8000	0.8000
F1 Score	0.8421	0.5263	0.6250	0.7368	0.8571
AUC	0.8500	0.5499	0.7000	0.7500	0.8500
Confusion Matrix	[9 1]<br>[2 8]	[6 4]<br>[5 5]	[9 1]<br>[5 5]	[8 2]<br>[3 7]	[8 2]<br>[1 9]
